# Hypoxia-induced Fascin-1 upregulation is regulated by Akt/Rac1 axis and enhances malignant properties of liver cancer cells via mediating actin cytoskeleton rearrangement and Hippo/YAP activation

**DOI:** 10.1038/s41420-021-00778-5

**Published:** 2021-12-11

**Authors:** Jian Pu, Youguan Huang, Quan Fang, Jianchu Wang, Wenchuan Li, Zuoming Xu, Xianjian Wu, Yuan Lu, Huamei Wei

**Affiliations:** 1 Department of Hepatobiliary Surgery, Affiliated Hospital of Youjiang Medical University for Nationalities, Baise, 533000 Guangxi China; 2 Graduate College of Youjiang Medical University for Nationalities, Baise, 533000 Guangxi China; 3 Department of Pathology, Affiliated Hospital of Youjiang Medical University for Nationalities, Baise, 533000 Guangxi China

**Keywords:** Hepatocellular carcinoma, Cancer prevention

## Abstract

In solid tumors, hypoxia facilitates malignant progression of cancer cells by triggering epithelial-mesenchymal transition (EMT) and cancer stemness. Fascin-1, an actin-bundling protein, takes part in the formation of many actin-based cellular structures. In the present study, we explored the potential functions of hypoxia-induced upregulation of Fascin-1 in liver cancer. Transcriptome RNA-sequencing was conducted to identify hypoxia-related genes. The potential functions of Fascin-1 were evaluated by western blot, transwell migration and invasion assays, sphere-formation assay, tumor xenograft growth, gelatin zymography analysis, immunofluorescence, cell viability assay, soft agar assay, and flow cytometry. We found that Fascin-1 was upregulated by hypoxia in liver cancer cell lines, elevated in liver cancer patients and correlated with larger tumor size, lymph node metastasis, distant metastasis, and shorter overall survival. Knockdown of Fascin-1 suppressed migration, invasion, EMT, stemness, and tumor xenograft growth of liver cancer cells under both normoxia and hypoxia conditions, while forced Fascin-1 expression showed opposite effects. Moreover, hypoxia-induced upregulation of Fascin-1 was regulated by the Akt/Rac1 signaling, and inhibition of Akt/Rac1 signaling by EHop-016 and MK-2206 restrained migration, invasion, EMT, and stemness of liver cancer cells under hypoxia. Furthermore, Fascin-1 knockdown suppressed MMP-2 and MMP-9 expression, impaired actin cytoskeleton rearrangement, inactivated Hippo/YAP signaling, and increased Sorafenib sensitivity in liver cancer cells. Our study provided a novel insight of Fascin-1 in regulating migration, invasion, EMT, and stemness of liver cancer cells under normoxia and hypoxia conditions.

## Introduction

Liver cancer is the second leading cause of cancer-related death worldwide and one of few cancers whose incidence and mortality are steadily increasing [1, 2]. Most of primary liver cancer cases are hepatocellular carcinoma (HCC; ≥90% all cases), with approximately 800,000 new diagnosed cases each year all over the world [3]. The prognosis for liver cancer patients is really dismal due to late stage at diagnosis, refractory to current therapy and high rate of recurrence. The 5-year overall survival of liver cancer patients remains low at 18%, and the recurrence rate is more than 50% at 5 years post-surgery [4]. It is important to understand the molecular carcinogenesis of liver cancer, which might help to develop novel therapeutic targets and prognostic markers.

Intratumoral hypoxia is a common phenomenon in solid tumors. In addition, hypoxia can promote migration, invasion, epithelial-mesenchymal transition (EMT), and stemness properties of cancer cells, thus increases the metastasis potential and survival of cells under hypoxia [5, 6]. Hypoxia-inducible factors (HIF-1α and HIF-2α) are two key transcriptional regulators induced by hypoxia. In HCC, hypoxia promotes stemness and tumorigenesis of HCC cells via SENP1-mediated HIF-1α deSUMOylation [7]. In the present study, we explored the potential genes that might involve in hypoxia-induced EMT and stemness of liver cancer cells.

Fascin-1 is a 55 kDa globular actin-bundling protein that creates cross-link between 10 and 30 parallel actin filaments [8]. By specifically interacting with F-actin, Fascin-1 takes part in the formation of many actin-based cellular structures, such as filopodia, lamellipodia, and microspikes [9, 10]. Besides, Fascin-1 also participates in the stabilization of mitochondrial and nuclear actin [11]. Fascin-1 is reported to facilitate migration, invasion, and EMT of cancer cells [12–14]. Dysregulation of Facin-1 has been found in liver cancer and participates in invasiveness, EMT and doxorubicin resistance [13, 15]. In the present study, we found that Fascin-1 was upregulated by hypoxia in liver cancer cells. The potential function of hypoxia-induced Fascin-1 was explored. Our study provided a novel insight of Fascin-1 in regulating migration, invasion, EMT, and stemness of liver cancer cells under normoxia and hypoxia conditions.

## Results

### Fascin-1 is upregulated by hypoxia in liver cancer cells and predicts poor prognosis of liver cancer patients

**Fig. 1:**
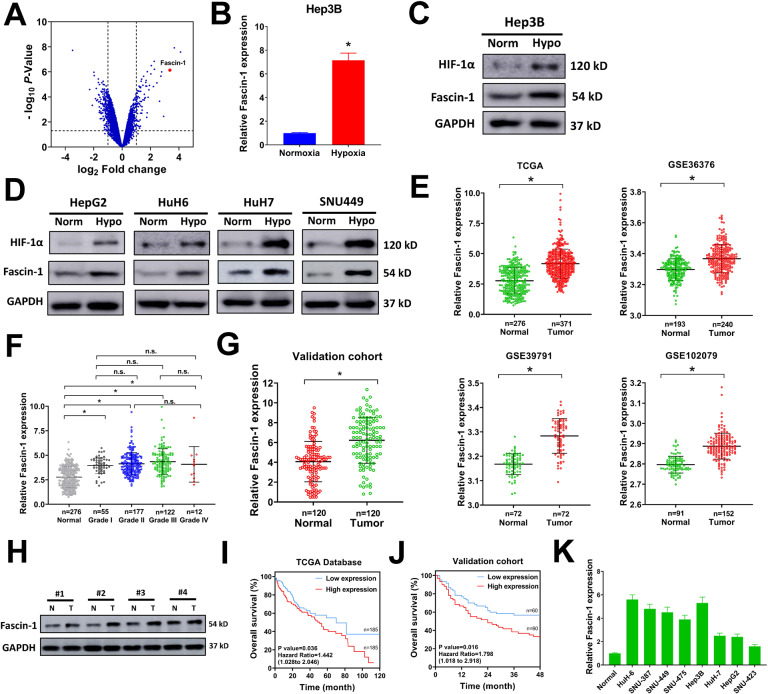
Fascin-1 is upregulated in liver cancer cells under hypoxia and predicts poor prognosis of liver cancer patients. **A** The differentially expressed genes in Hep3B cells exposed to hypoxia versus normoxia were depicted in volcano plot. **B** Hep3B cells were exposed to normoxia or hypoxia condition for 24 h, then relative Fascin-1 expression was evaluated by qRT-PCR. **C** Hep3B cells were exposed to normoxia or hypoxia condition for 24 h, then protein expression of Fascin-1 and HIF-1α were evaluated by western blot. **D** HepG2, HuH-6, HuH-7 and SNU-449 cells were exposed to normoxia or hypoxia condition for 24 h, then protein expression of Fascin-1 and HIF-1α were evaluated by western blot. **E** Relative Fascin-1 expression in liver cancer tissues and paired normal samples of TCGA and GEO (GSE36376, GSE39791, and GSE102079) database was shown. **F** Relative Fascin-1 expression in TCGA-liver cancer subset according to tumor stage. **G** Relative Fascin-1 expression in 120 pairs of liver cancer tissues and paired adjacent normal tissues of the validation cohort was evaluated by qRT-PCR. **H** Fascin-1 expression in liver cancer patients and paired adjacent normal tissues of the validation cohort was evaluated by western blot. **I**, **J** Kaplan-Meier survival analysis of liver cancer patients according to Fascin-1 expression in TCGA (**I**) and the validation cohort (**J**). **K** Relative Fascin-1 expression in liver cancer cell lines was evaluated by qRT-PCR. Norm, normoxia condition; Hypo, hypoxia condition. n.s. = not significant. ^*^P < 0.05.

To explore the potential gene that involve in regulating hypoxia-induced malignant properties of liver cancer, a liver cancer cell line Hep3B was exposed to normoxia (20% O_2_) or hypoxia (1% O_2_) conditions, then dysregulated genes were evaluated by transcriptome RNA-sequencing. The dysregulated genes of Hep3B cells under hypoxia condition compared with normoxia condition were depicted in the volcano map (Fig. 1A). A total of 78 upregulated genes and 150 downregulated genes were discovered (Supplementary Table 1). KEEG and GO analysis found that HIF-1 signaling pathway was significantly activated while RNA transport and ncRNA metabolic process were significantly restrained (Supplementary Fig. 1). In our study, we focused on Fascin-1 because Fascin-1 ranked the top 5 upregulated genes in Hep3B cells under hypoxia (Fig. 1A and Supplementary Table 1), and previous studies indicated that Fascin-1 is dysregulated in liver cancer patients and associates with distant metastasis, recurrence, and poor survival [16–18]. The expression level of Fascin-1 in Hep3B cells under hypoxia was further validated by quantitative real-time polymerase chain reaction (qRT-PCR) and western blot. The mRNA and protein levels of Fascin-1 were significantly increased in Hep3B cells under hypoxia condition (Fig. 1B, C). HIF-1α is a key transcriptional regulator induced by hypoxia. The level of HIF-1α was evidently upregulated in Hep3B cells under hypoxia, too (Fig. 1C). Moreover, Fascin-1 was also upregulated under hypoxia in a list of tested liver cancer cells, indicating that hypoxia-induced upregulation of Fascin-1 was common in liver cancer cell lines (Fig. 1D).

As previous studies reported that Fascin-1 was upregulated in liver cancer patients [16–18], we searched for the Fascin-1 expression values in The Cancer Genome Atlas (TCGA) database and Gene Expression Omnibus (GEO) database. Fascin-1 was apparently increased in tumor tissues of liver cancer patients, as observed in TCGA-HCC cohort, GSE36376, GSE39791, and GSE102079 (Fig. 1E). We also compared the expression levels of Fascin-1 in liver cancer patients of different tumor stages in TCGA database. However, the expression of Fascin-1 had no connection with tumor stage, suggesting that upregulation of Fascin-1 in liver cancer patients might be an early event (Fig. 1F). The upregulation of Fascin-1 was tested in a validation cohort of liver cancer patients in our study, and we found that Fascin-1 was significantly upregulated in liver cancer tissues, too (Fig. 1G). The elevated Fascin-1 expression in the validation cohort was also verified by western blot. The protein level of Fascin-1 was increased in tumor tissues of liver cancer patients (Fig. 1H). Furthermore, the connection between Fascin-1 expression and clinicopathological characteristics of liver cancer patients was evaluated in the validation cohort. High Fascin-1 expression was positively correlated with larger tumor size, lymph node metastasis, and distant metastasis (Table 1). No connection between Fascin-1 expression and tumor stage was found, too (Table 1). In Kaplan-Meier survival analysis, high Fascin-1 expression was positively associated with poor overall survival of liver cancer patients in TCGA database and our validation cohort (Fig. 1I, J). In addition, Fascin-1 was high expressed in HuH-6 and Hep3B cells, and low expressed in HuH-7, HepG2, and SNU-423 cells (Fig. 1K). Taken together, our results indicated that Fascin-1 was upregulated by hypoxia in liver cancer cells, elevated in liver cancer patients, and associated with poor prognosis.

**Table 1:** Association between Fascin-1 expression and clinicopathological characteristics in liver cancer patients.

Characteristics	*n*	Fascin-1 expression		*P* value
		Low	High	
Age (years)				0.178
≤60	41	24	17	
>60	79	36	43	
Sex				0.269
Male	68	31	37	
Female	52	29	23	
Tumor size (mm)				<0.001
≥30	89	36	53	
<30	31	24	7	
TNM stage				0.487
I–II	23	13	10	
III–IV	97	47	50	
Lymph node metastasis				0.009
Positive	85	36	49	
Negative	35	24	11	
Distant metastasis				<0.001
Positive	46	11	35	
Negative	74	49	25	

### Knockdown of Fascin-1 suppresses migration, invasion, EMT, stemness, and tumor xenograft growth of liver cancer cells

**Fig. 2:**
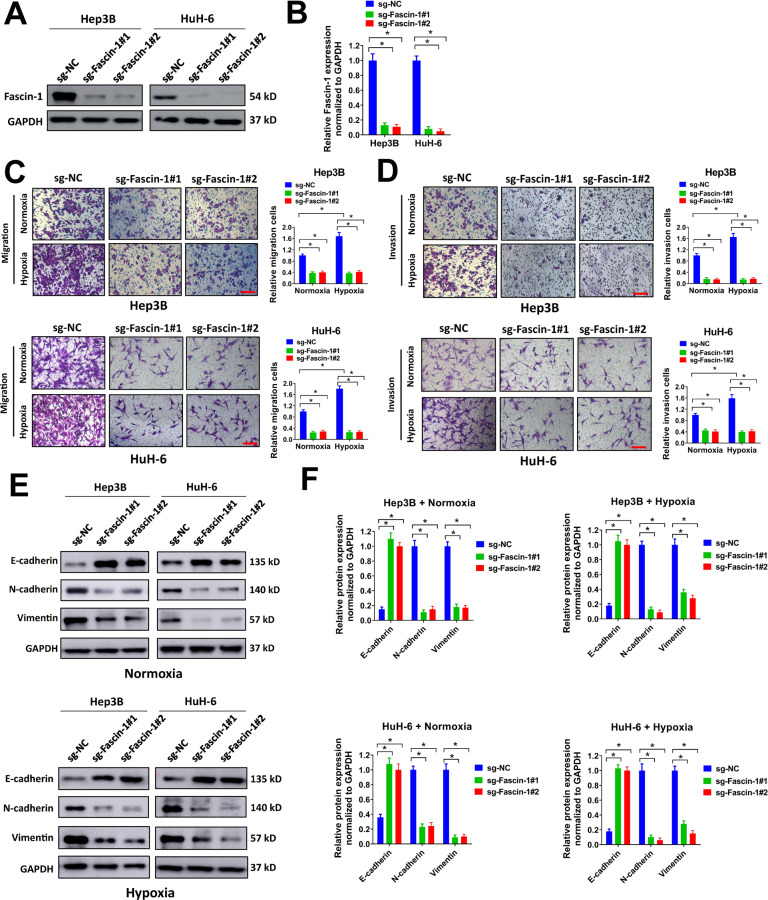
Knockdown of Fascin-1 suppresses migration, invasion, and EMT of liver cancer cells. **A**, **B** Hep3B or HuH-6 cells infected with sg-Fascin-1#1, sg-Fascin-1#2, or sg-NC lentivirus were used for western blot (**A**). Relative Fascin-1 expression normalized to GAPDH was shown (**B**). **C**, **D** Hep3B or HuH-6 cells (2.5 × 10^4^) infected with sg-Fascin-1#1, sg-Fascin-1#2, or sg-NC lentivirus were seeded in transwell chamber for transwell migration (**C**) or invasion (**D**) assay under normoxia or hypoxia condition. Relative migration (**C**) or invasion (**D**) cells were shown. Scale bar = 100 μm. **E**, **F** Hep3B or HuH-6 cells infected with sg-Fascin-1#1, sg-Fascin-1#2, or sg-NC lentivirus were exposed to normoxia or hypoxia condition for 72 h, then cell lysates were collected for western blot (**E**). Relative expression of E-cadherin, N-cadherin, and Vimentin were normalized to GAPDH (**F**). ^*^P < 0.05.

**Fig. 3:**
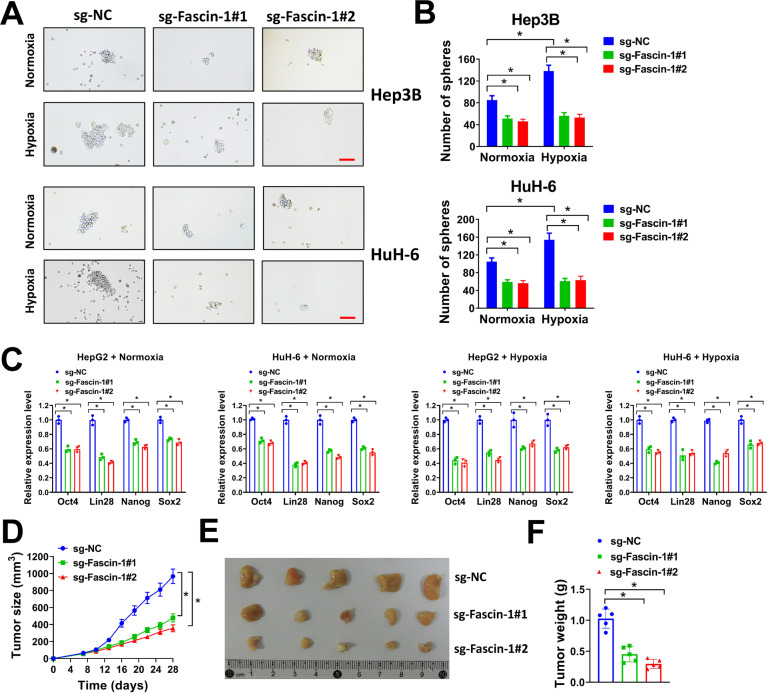
Knockdown of Fascin-1 inhibits stemness and tumor xenograft growth of liver cancer cells. **A**, **B** Hep3B or HuH-6 cells (2000/well) infected with sg-Fascin-1#1, sg-Fascin-1#2, or sg-NC lentivirus were seeded in 6-well ultra-low attachment plates for sphere-formation assay under normoxia or hypoxia condition (**A**). Number of spheres per well was shown (**B**). Scale bar = 500 μm. **C** Hep3B or HuH-6 cells infected with sg-Fascin-1#1, sg-Fascin-1#2, or sg-NC lentivirus were exposed to normoxia or hypoxia condition for 72 h, then relative expression of Oct4, Lin28, Nanog, and Sox2 were evaluated by qRT-PCR. **D**–**F** Hep3B (2 × 10^6^ cells) introduced with sg-Fascin-1#1, sg-Fascin-1#2, or sg-NC were subcutaneously injected into nude mice and tumor xenografts were allowed to grow for 4 weeks. Tumor growth curve (**D**), representative image (**E**), and tumor weight (**F**) were shown. ^*^P < 0.05.

To explore the potential function of hypoxia-induced upregulation of Fascin-1 in liver cancer, we constructed two single guide RNAs (sgRNAs) (sg-Fascin-1#1 and sg-Fascin-1#2) specifically targeting Fascin-1 and depleted Fascin-1 expression using the CRISPR/Cas9 system. Hep3B and HuH-6 cells were selected due to their high endogenous expression levels of Fascin-1 (Fig. 1K). In western blot, Fascin-1 was successfully knocked down in Hep3B and HuH-6 cells by sg-Fascin-1#1 or sg-Fascin-1#2 (Fig. 2A). As hypoxia promotes migration, invasion, EMT, and stemness of cancer cells [5, 19, 20], we evaluated the influence of Fascin-1 knockdown on these properties of liver cancer cells under normoxia or hypoxia conditions. In our study, Fascin-1 knockdown impaired transwell migration and invasion of Hep3B and HuH-6 cells under both normoxia and hypoxia conditions (Fig. 2C, D). In addition, it was worth noting that under hypoxia condition, the number of migration and invasion cells of Hep3B and HuH-6 was apparently increased compared with these under normoxia condition, suggesting that hypoxia promoted migration and invasion of Hep3B and HuH-6 cells (Fig. 2C, D). However, these effects were abrogated by Fascin-1 knockdown, as there was no apparent difference in the number of migration or invasion cells under normoxia and hypoxia conditions after Fascin-1 knockdown (Fig. 2C, D). To evaluate the influence of Fascin-1 knockdown on EMT of liver cancer cells, the levels of EMT markers (E-cadherin, N-cadherin, and Vimentin) were evaluated by western blot. Fascin-1 knockdown elevated E-cadherin expression, and restrained N-cadherin and Vimetin expression in Hep3B and HuH-6 cells under both normoxia and hypoxia conditions, indicating that Fascin-1 knockdown repressed EMT (Fig. 2E, F). In sphere-formation assay, depletion of Fascin-1 dramatically reduced the number of spheres formed by Hep3B and HuH-6 cells under both normoxia and hypoxia conditions (Fig. 3A, B). Meanwhile, hypoxia increased the number of sphere formed by Hep3B and HuH-6 cells compared with normoxia condition, but this was abolished by Fascin-1 knockdown (Fig. 3A, B). In qRT-PCR analysis, the expression levels of stemness markers (Oct4, Lin28, Nanog, and Sox2) were also downregulated by Fascin-1 knockdown in Hep3B and HuH-6 cells (Fig. 3C). We also evaluated the influence of Fascin-1 knockdown on tumor xenograft growth of Hep3B cells in nude mice. We found that Fascin-1 knockdown suppressed tumor xenograft growth and reduced tumor volume and weight (Fig. 3D–F). Collectively, our results indicated that Fascin-1 knockdown suppressed migration, invasion, EMT, stemness, and tumor xenograft growth of liver cancer cells under both normoxia and hypoxia conditions, and abrogated the effects caused by hypoxia on migration, invasion, and stemness of liver cancer cells.

### Forced Fascin-1 expression promotes migration, invasion, EMT, stemness, and tumor xenograft growth of liver cancer cells

**Fig. 4:**
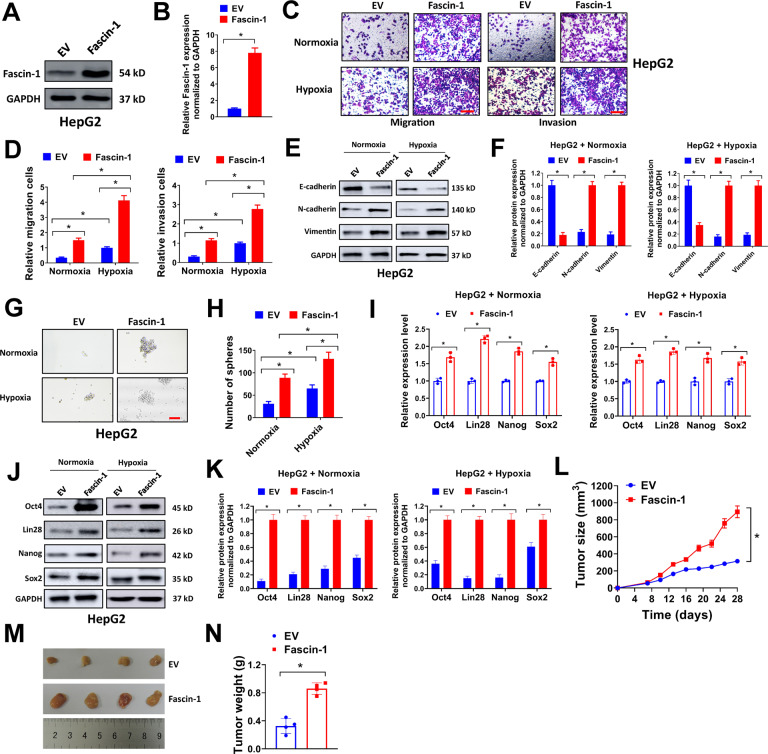
Forced Fascin-1 expression promotes migration, invasion, EMT, stemness, and tumor xenograft growth of liver cancer cells. **A**, **B** HepG2 cells were infected with Fascin-1 expression lentivirus or empty vector (EV) control, then lysates were collected for western blot (**A**). Relative Fascin-1 expression normalized to GAPDH was shown (**B**). **C**, **D** HepG2 cells (2.5 × 10^4^) infected with Fascin-1 expression lentivirus or EV control were seeded in transwell chamber for transwell migration (**C**) or invasion (**D**) assay under normoxia or hypoxia condition. Relative migration (**C**) or invasion (**D**) cells were shown. Scale bar = 100 μm. **E**, **F** HepG2 cells infected with Fascin-1 expression lentivirus or EV control were exposed to normoxia or hypoxia condition for 72 h, then cell lysates were collected for western blot (**E**). Relative expression of E-cadherin, N-cadherin, and Vimentin were normalized to GAPDH (**F**). **G**, **H** HepG2 cells (2000/well) infected with Fascin-1 expression lentivirus or EV control were seeded in 6-well ultra-low attachment plates for sphere-formation assay under normoxia or hypoxia condition (**G**). Number of spheres per well was shown (**H**). Scale bar = 500 μm. **I**–**K** HepG2 cells infected with Fascin-1 expression lentivirus or EV control were exposed to normoxia or hypoxia condition for 72 h, then relative expression of Oct4, Lin28, Nanog, and Sox2 were evaluated by qRT-PCR (**I**), or collected cell lysates for western blot (**J**). Relative expression of E-cadherin, N-cadherin, and Vimentin were normalized to GAPDH (**K**). **L**–**N** HepG2 (2 × 10^6^ cells) infected with Fascin-1 expression lentivirus or EV control were subcutaneously injected into nude mice and tumor xenografts were allowed to grow for 4 weeks. Tumor growth curve (**L**), representative image (**M**), and tumor weight (**N**) were shown. ^*^P < 0.05.

The potential function of Fascin-1 in live cancer was also evaluated by gain-of-function assays. In our study, a Fascin-1 expression lentivirus vector was constructed and introduced into HepG2 cells, which had low endogenous level of Fascin-1. As shown in Fig. 4A, B, we successfully overexpressed Fascin-1 in HepG2 cells. Next, enforced Fascin-1 expression increased the number of migration and invasion cells in HepG2 under both normoxia and hypoxia conditions (Fig. 4C, D). In western blot analysis, overexpression of Fascin-1 reduced the expression of E-cadherin and increased the expression of N-cadherin and Vimentin in HepG2 cells, indicating that forced Fascin-1 expression promoted EMT of liver cancer cells under both normoxia and hypoxia conditions (Fig. 4E, F). In addition, forced Fascin-1 expression also facilitated sphere formation of HepG2 cells (Fig. 4G, H) and increased the mRNA and protein expression levels of stemness markers Oct4, Lin28, Nanog, and Sox2 (Fig. 4I–K). The influence of Fascin-1 overexpression on tumor xenograft growth was also tested. Forced Fascin-1 expression enhanced tumor xenograft growth of HepG2 cells, with increased tumor volume and weight (Fig. 4L–N). Above all, our results indicated that forced Fascin-1 expression promoted migration, invasion, EMT, stemness, and tumor xenograft growth of liver cancer cells.

### Hypoxia-induced activation of Akt/Rac1 signaling increases Fascin-1 expression and regulates migration, invasion, EMT, and stemness of liver cancer cells

**Fig. 5:**
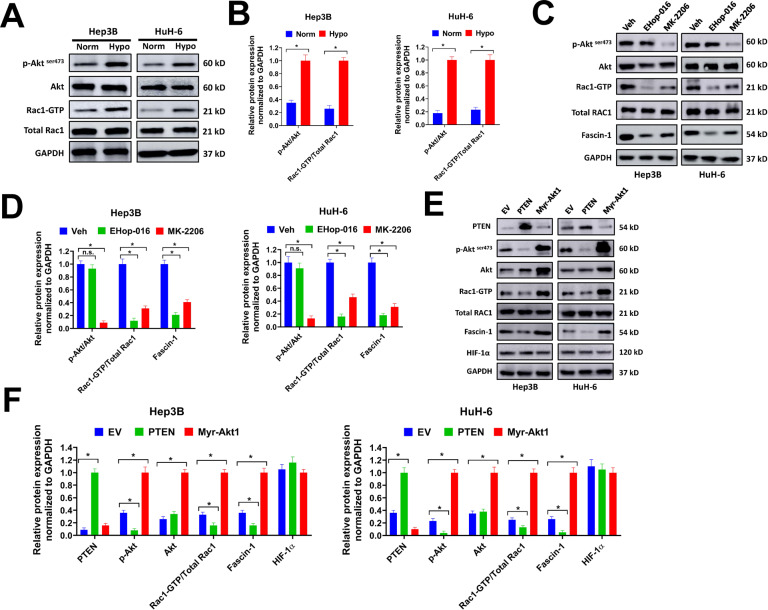
Hypoxia-induced activation of Akt/Rac1 signaling increases Fascin-1 expression in liver cancer cells. **A**, **B** Hep3B or HuH-6 cells were exposed to normoxia or hypoxia condition for 72 h, then cell lysates were collected for western blot (**A**). Relative protein expression was normalized to GAPDH (**B**). **C**, **D** Hep3B and HuH-6 cells (1 × 10^6^ cells) were seeded in 6-well plates, then treated with EHop-016 (1 μM), MK-2206 (1 μM), or equal volume of DMSO (Veh) for 24 h under hypoxia condition. Collected cell lysates for western blot (**C**). Relative protein expression normalized to GAPDH (**D**) was shown. **E**, **F** Hep3B or HuH-6 cells infected with PTEN, Myr-Akt1, or EV lentivirus were exposed to hypoxia for 72 h, then cell lysates were collected for western blot (**E**). Relative protein expression normalized to GAPDH (**F**) was shown. Norm, normoxia condition; Hypo, hypoxia condition. n.s. = not significant. ^*^P < 0.05.

**Fig. 6:**
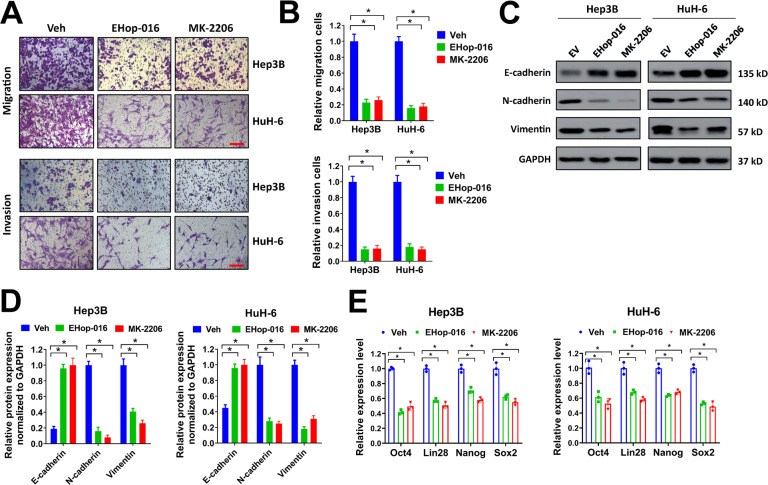
Inhibition of Akt/Rac1 signaling restrains migration, invasion, EMT, and stemness of liver cancer cells under hypoxia. **A**, **B** Hep3B or HuH-6 cells (2.5 × 10^4^) were seeded in transwell chamber for transwell migration or invasion assay under hypoxia condition (**A**). Cells were treated with EHop-016 (1 μM), MK-2206 (1 μM), or equal volume of DMSO (Veh) at the same time. Relative migration or invasion cells were shown (**B**). Scale bar = 100 μm. **C**–**E** Hep3B and HuH-6 cells (1 × 10^6^ cells) were seeded in 6-well plates, then treated with EHop-016 (1 μM), MK-2206 (1 μM), or equal volume of DMSO (Veh) for 72 h under hypoxia condition. Collected cell lysates for western blot (**C**, **D**) or extracted total RNAs for qRT-PCR. Relative expression of E-cadherin, N-cadherin, and Vimentin were normalized to GAPDH (**D**). Relative expression of Oct4, Lin28, Nanog, and Sox2 were shown (**F**). *P < 0.05.

The HIF-1 signaling pathway plays a crucial role for tumor cells to adapt to hypoxia [5, 19, 20]. In KEEG pathway analysis, HIF-1α signaling was activated in Hep3B cells under hypoxia (Supplementary Fig. 1). As hypoxia induced upregulation of Fascin-1 in liver cancer cells, we speculated that the HIF-1α signaling might regulate Fasin-1 expression to some extent. Therefore, two sgRNAs specifically targeting HIF-1α (sg-HIF-1α#1 and sg-HIF-1α#2) were designed and depleted HIF-1α expression in Hep3B and HuH-6 cells (Supplementary Fig. 2A, B). However, knockdown of HIF-1α showed no influence on Fascin-1 expression, despite that the migration and invasion of Hep3B and HuH-6 cells were seriously impaired (Supplementary Fig. 2C, D). These results indicated that hypoxia-induced Fascin-1 upregulation was not regulated by HIF-1α pathway. In previous reports, Fascin-1 was proved to be regulated by Rho family GTPases Rac1 [21, 22]. In our study, the levels of p-Akt ^ser473^ and Rac1-GTP was significantly increased in Hep3B and HuH-6 cells under hypoxia condition, indicating that hypoxia promoted the activation of Akt and Rac1 (Fig. 5A, B). To test if the activation of Akt and Rac1 was involved in hypoxia-induced upregulation of Fascin-1, we used EHop-016 (Rac1 inhibitor) and MK-2206 (Akt inhibitor) to suppress Akt and Rac1 activation in liver cancer cells. Both the Rac1 inhibitor EHop-016 and Akt inhibitor MK-2206 reduced the expression level of Fascin-1 in Hep3B and HuH-6 cells under hypoxia condition (Fig. 5C, D). Moreover, MK-2206 reduced the levels of p-Akt ^ser473^ and Rac1-GTP, while EHop-016 showed no influence on the level of p-Akt ^ser473^, indicating that Akt acted upstream of Rac1. To further evaluate the influence of Akt/Rac1 axis on hypoxia-induced Fascin-1, we forced expression of PTEN or Myr-Akt1 in liver cancer cells. PTEN is a negative regulator of PI3K/Akt signaling, while Myr-Akt1 is a constitutively active form of Akt1. In our study, overexpression of PTEN evidently reduced the expression levels of p-Akt ^ser473^, Rac1-GTP, and Fascin-1 in Hep3B and HuH-6 cells under hypoxia condition (Fig. 5E, F). In contrast, Myr-Akt1 obviously increased the expression levels of p-Akt ^ser473^, Rac1-GTP, and Fascin-1 (Fig. 5E, F). Moreover, overexpression of PTEN or Myr-Akt1 showed no apparent influence on the protein level of HIF-1α in Hep3B and HuH-6 cells under hypoxia condition. Next, we evaluated the influence of Akt/Rac1 signaling on migration, invasion, EMT, and stemness of liver cancer cells. In transwell migration and invasion assays, EHop-016 and MK-2206 suppressed migration and invasion of Hep3B and HuH-6 cells under hypoxia (Fig. 6A, B). Meanwhile, EHop-016 and MK-2206 increased the expression of E-cadherin and suppressed the expression of N-cadherin and Vimentin, indicating that EHop-016 and MK-2206 restrained EMT of Hep3B and HuH-6 cells under hypoxia (Fig. 6C, D). In addition, EHop-016 and MK-2206 treatment reduced the expression of stemness markers Oct4, Lin28, Nanog, and Sox2 (Fig. 6E). Taken together, our results indicated that hypoxia-induced activation of Akt/Rac1 signaling increased Fascin-1 expression and regulated migration, invasion, EMT, and stemness of liver cancer cells under hypoxia.

### Fascin-1 regulates actin cytoskeleton rearrangement and Hippo/YAP activation in liver cancer cells

**Fig. 7:**
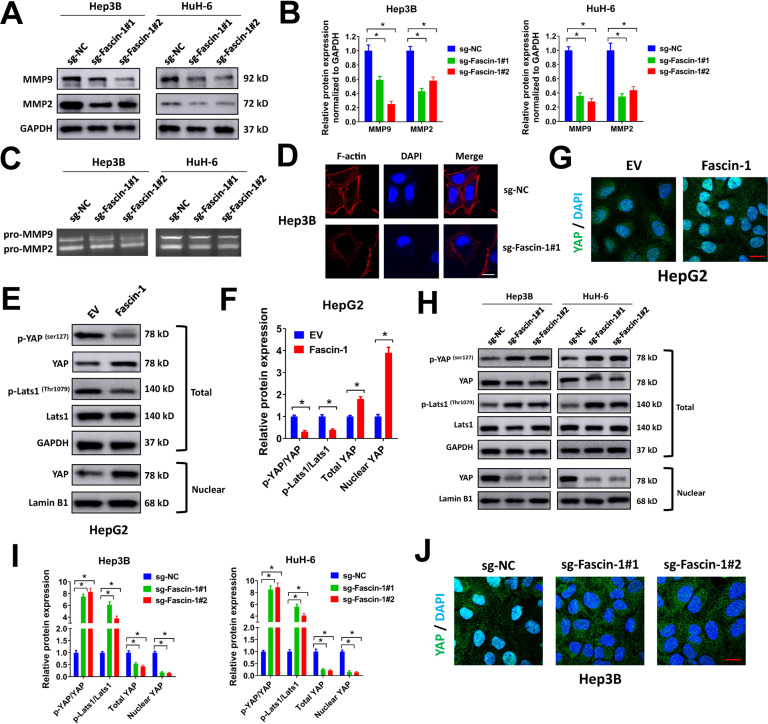
Fascin-1 regulates actin cytoskeleton rearrangement and Hippo/YAP activation in liver cancer cells. **A**, **B** Hep3B or HuH-6 cells were infected with sg-Fascin-1#1, sg-Fascin-1#2, or sg-NC lentivirus, then cell lysates were collected for western blot (**A**). Relative MMP-2 or MMP-9 expression normalized to GAPDH was shown (**B**). **C** Hep3B or HuH-6 cells were infected with sg-Fascin-1#1, sg-Fascin-1#2, or sg-NC lentivirus, then conditioned medium were collected for gelatin zymography analysis. **D** Hep3B cells were infected with sg-Fascin-1#1 or sg-NC lentivirus, then stained with Alexa Fluor^®^ 555 Phalloidin and DAPI for immunofluorescence. Scale bar = 10 μm. **E**, **F** HepG2 cells were infected with Fascin-1 lentivirus or EV control, then lysates were collected for western blot (**E**). Relative protein expression was shown (**F**). **G** HepG2 cells were infected with Fascin-1 lentivirus or EV control, then stained with Alexa Fluor^®^ 488 Conjugated YAP Rabbit mAb and DAPI for immunofluorescence. Scale bar = 10 μm. **H**, **I** Hep3B or HuH-6 cells were infected with sg-Fascin-1#1, sg-Fascin-1#2, or sg-NC lentivirus, then lysates were collected for western blot (**H**). Relative protein expression was shown (**I**). **J** Hep3B cells were infected with sg-Fascin-1#1, sg-Fascin-1#2, or sg-NC lentivirus, then stained with Alexa Fluor^®^ 488 Conjugated YAP Rabbit mAb and DAPI for immunofluorescence. Scale bar = 10 μm. ^*^P < 0.05.

In previous studies, Fascin-1 is reported to regulate migration and invasion of cancer cells via upregulating matrix metalloproteinases (MMP) expression, such as MMP2 and MMP9 [12, 13, 23]. In our study, we also evaluated the influence of Fascin-1 on MMP2 and MMP9 expression in liver cancer cells. In western blot analysis, knockdown of Fascin-1 reduced the protein expression of MMP2 and MMP9 in Hep3B and HuH-6 cells (Fig. 7A, B). In gelatin zymographic analysis, the levels of pro-MMP2 and pro-MMP9 in conditioned media of Hep3B and HuH-6 cells were decreased by Fascin-1 knockdown (Fig. 7C). These results were corresponding with previous reports. As actin-bundling protein, Fascin-1 might play a role in actin cytoskeleton dynamics. As we expected, phalloidin staining of actin filaments revealed that depletion of Fascin-1 impaired actin cytoskeleton rearrangement in Hep3B cells (Fig. 7D). We also evaluated the impact of Facin-1 on downstream signaling pathways. Unlike other conventional signaling pathways, Hippo-YAP signaling is impacted and activated by architectural and mechanical cues, and actin cytoskeleton is an important mediator of Hippo-YAP signaling [24]. Thus, we evaluated the influence of Fascin-1 on Hippo-YAP signaling. In our study, we found that forced expression of Fascin-1 reduced the phosphorylation of YAP and Lats1 and increased the total and nuclear levels of YAP in HepG2 cells, indicating that Fascin-1 overexpression promoted Hippo-YAP activation in liver cancer cells (Fig. 7E, F). In addition, immunofluorescence staining of YAP revealed that Fascin-1 overexpression increased nuclear translocation of YAP in HepG2 cells (Fig. 7G). On the contrary, knockdown of Fascin-1 increased the phosphorylation of YAP and Lats1 and decreased the total and nuclear levels of YAP in Hep3B and HuH-6 cells, suggesting that Fascin-1 knockdown suppressed Hippo-YAP activation in liver cancer cells (Fig. 7H, I). Moreover, we demonstrated that Fascin-1 knockdown reduced nuclear translocation of YAP in Hep3B cells by immunofluorescence staining (Fig. 7J). Above all, our results indicated that Fascin-1 regulated actin cytoskeleton rearrangement and Hippo/YAP activation in liver cancer cells.

### Knockdown of Fascin-1 increases Sorafenib sensitivity of liver cancer cells

**Fig. 8:**
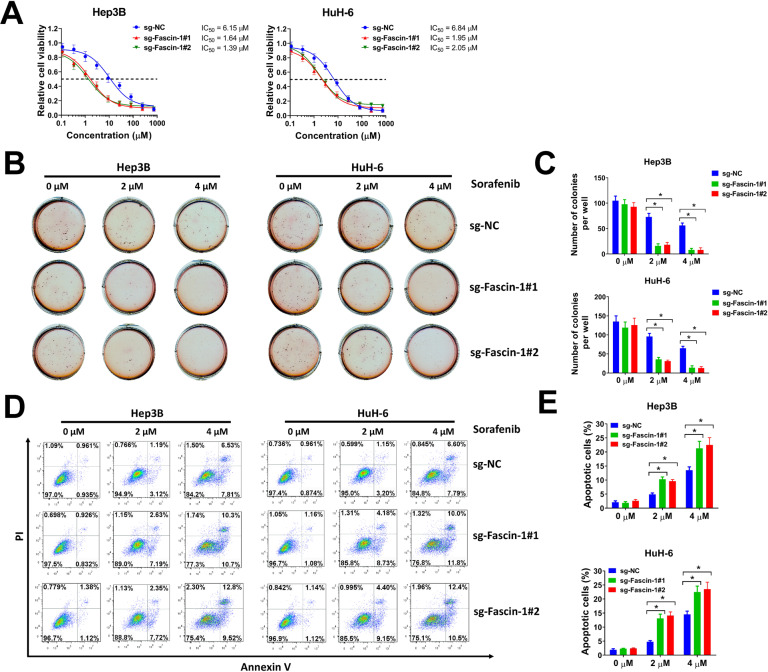
Knockdown of Fascin-1 increases Sorafenib sensitivity of liver cancer cells. **A** Hep3B or HuH-6 cells (3000/well) infected with sg-Fascin-1#1, sg-Fascin-1#2, or sg-NC lentivirus were seeded in 96-well plates, and treated with 0, 0.11, 0.33, 1, 3, 9, 27, 81, 243, and 729 μM Sorafenib for 6 days, then assayed for cell viability. **B**, **C** Hep3B or HuH-6 cells (6000/well) infected with sg-Fascin-1#1, sg-Fascin-1#2, or sg-NC lentivirus were used for soft agar colony-formation assay. Cells were treated with 0, 2, or 4 μM Sorafenib at the same time. Representative plates (**B**) and number of colonies per well (**C**) were shown. **D**, **E** Hep3B or HuH-6 cells (1 × 10^6^ cells) infected with sg-Fascin-1#1, sg-Fascin-1#2, or sg-NC lentivirus were seeded in 6-well plates and treated with 0, 2, or 4 μM Sorafenib for 72 h, then cells were stained with Annexin V-FITC and PI for flow cytometry (**D**). The percentage of apoptotic cells were shown (**E**). ^*^P < 0.05.

In the present study, we found that Fascin-1 promoted EMT and stemness of liver cancer cells under both normoxia and hypoxia conditions. As numerous reports prove that EMT and cancer stemness might contribute to drug resistance [20, 25], we speculated that Fascin-1 might affect drug sensitivity of liver cancer cells in some degree. The multikinase inhibitor Sorafenib is the first drug approved for the treatment of advanced liver cancer. In our study, we evaluated the influence of Fascin-1 knockdown on drug sensitivity of Sorafenib in liver cancer cells. As shown in Fig. 8A, knockdown of Fascin-1 reduced the IC_50_ of Sorafenib in both Hep3B and HuH-6 cells compared with sg-NC group. In soft agar assay, Sorafenib (2 and 4 μM) showed moderate suppression on colony formation of Hep3B and HuH-6 cells, and knockdown of Fascin-1 significantly enhanced the inhibitory effect of Sorafenib compared with sg-NC group (Fig. 8B, C). In flow cytometry, the percentage of apoptotic cells were increased by Sorafenib (2 and 4 μM) treatment in sg-NC group, and this effect was dramatically enhanced by Fascin-1 knockdown in Hep3B and HuH-6 cells (Fig. 8D, E). Collectively, our results indicated that knockdown of Fascin-1 increased Sorafenib sensitivity in liver cancer cells.

## Discussion

In the present study, we found that Fascin-1 was significantly upregulated under hypoxia condition in liver cancer cells. Meanwhile, Fascin-1 was elevated in liver cancer patients and predicted poor prognosis. Indeed, several previous studies report that Fascin-1 is elevated in tumor tissues of liver cancer patients, and correlates with larger tumor size, lymph node metastasis, distant metastasis, and poor overall survival [16–18]. In our study, we also found that high Fascin-1 expression was positively correlated with increased tumor size, lymph node metastasis, distant metastasis, and shorter overall survival. The potential function of Fascin-1 in liver cancer has been studied by others, too. Yoshihiro Hayashi et al. found that Fascin-1 promoted invasiveness of HCC cells by reducing E-cadherin expression and increasing MMP2 and MMP9 secretion [13]. Yuanbiao Zhang et al. found that Fascin-1 increased doxorubicin resistance of HCC cells by promoting EMT [15]. Similar with their findings, we proved that Fascin-1 knockdown decreased MMP2 and MMP9 expression, and increased Sorafenib sensitivity in liver cancer cells. In the present study, though we found that Fascin-1 and HIF-1α were both elevated under hypoxia, our results proved that knockdown of HIF-1α showed no apparent influence on Fascin-1 expression.

In the present study, we found that hypoxia-induced upregulation of Fascin-1 was regulated by the Akt/Rac1 axis. Rac1, a member of Rho family GTPases, plays an important role in formation of lamellipodia and membrane ruffles [26]. Rac1 is found to upregulate in liver cancer patients and predict poor prognosis [27]. Furthermore, Rac1 is proved to play a role in regulating Fascin-1 medicated formation of microspikes and lamellipodia [21]. In our study, we found that inhibition of Rac1 activity reduced hypoxia-mediated upregulation of Fascin-1 and suppressed migration, invasion, EMT, and stemness of liver cancer cells. Moreover, we found that Rac-1 activity was augmented by hypoxia-induced activation of Akt. Our data indicated that Akt was probably upstream of Rac1, and upregulation of phosphorylated Akt elevated the Rac1-GTP level. Regulating of Rac1 activity by Akt signaling has been reported by other studies [28]. It is worth noting that various studies prove that Akt signaling is activated under hypoxia condition in cancer cells [29, 30]. However, the Akt signaling is dispensable for HIF-1 activity, though this is context dependent [31]. Correspondingly, we found that overexpression of PTEN or Myr-Akt1 exhibited no influence on HIF-1α level in liver cancer cells.

In our study, we found that Fascin-1 knockdown impaired actin cytoskeleton rearrangement and suppressed Hippo/YAP signaling in liver cancer cells. As actin-bundling protein, Fascin-1 regulates actin dynamic in cell protrusions, filopodia, spikes, and lamellipodial ribs [8]. Thus, it is not a surprise that knockdown of Fascin-1 impaired actin cytoskeleton rearrangement in liver cancer cells in our study. This could also explain why Fascin-1 regulated migration and invasion of liver cancer cells. In addition, Fascin-1 specifically binds to F-actin, thus forms 10–30 parallel actin filaments. These actin bundles can transport signaling proteins from cell body to the very edge of the cell [32]. This indicates that Fascin-1 has the potential to effect the activation of signaling pathways. Indeed, we found that knockdown of Fascin-1 increased the phosphorylation of YAP, the key transcription co-activator of Hippo tumor suppressor pathway. It is proved that YAP activity is tightly regulated by actin cytoskeleton architecture [24]. Moreover, there are increasing evidences proving that Hippo/YAP signaling is involved in migration, invasion, EMT, stemness, and actin cytoskeleton remodeling of cancer cells [33]. Thus we speculated that Fascin-1 regulated migration, invasion, EMT, and stemness of liver cancer by regulating actin cytoskeleton rearrangement and Hippo/YAP activation, but more evidences were needed to further prove this.

In conclusion, we found that Fascin-1 was upregulated by hypoxia in liver cancer cells, elevated in liver cancer patients, and associated with poor prognosis. Knockdown of Fascin-1 suppressed migration, invasion, EMT, stemness, and tumor xenograft growth of liver cancer cells under both normoxia and hypoxia conditions, while forced Fascin-1 expression showed opposite effects. Moreover, we found that hypoxia-induced Fascin-1 upregulation was regulated by the Akt/Rac1 signaling. Inhibition of Akt/Rac1 suppressed migration, invasion, EMT, and stemness of liver cancer cells under hypoxia condition. Furthermore, we found that Fascin-1 knockdown impaired actin cytoskeleton rearrangement, inactivated Hippo/YAP signaling, and increased Sorafenib sensitivity. Our results demonstrated how hypoxia-induced upregulation of Fascin-1 enhanced migration, invasion, EMT, and stemness of liver cancer cells.

## Materials and methods

### Patient samples

A total of 120 liver cancer tissues and matched adjacent non-tumor tissues were collected from Affiliated Hospital of Youjiang Medical University for Nationalities between May 2016 and March 2017. Written informed consent was obtained from each enrolled patient. Use of human samples was reviewed and approved by the ethics committee of Affiliated Hospital of Youjiang Medical University for Nationalities. Tumor stage was determined by the TNM staging system. The demographic and clinicopathological data of enrolled patients was collected from patient records. All patients were followed up to 48 months post-surgery.

### Cell culture and reagents

Liver cancer cell lines SNU-387, SNU-449, SNU-475, Hep3B, HepG2 and SNU-423, and HEK293T cells were purchased from American Type Culture Collection (ATCC). Liver cancer cell lines HuH-6 and HuH-7 were purchased from RIkagaku KENkyusho/Institute of Physical and Chemical Research (RIKEN). All cell lines were cultured in RPMI-1640 medium (Gibco, USA) supplemented with 10% fetal bovine serum (Hyclone, USA) and 1% penicillin/streptomycin (Hyclone, USA) at 37 °C with 5% CO_2_ in a humidified atmosphere. To evaluate the potential influence of hypoxia, cells were cultured with 1.0% O_2_ in a hypoxic chamber (Thermo Fisher Scientific, USA). MK-2206 (Selleck #S1078) and EHop-016 (Selleck #S7319) were purchased from Selleck Chemicals (USA) and dissolved in DMSO for later use. Thus, DMSO was used as vehicle control.

### Plasmid constructs and lentivirus package

Fascin-1 and PTEN expression lentiviral plasmids were constructed by cloning the coding sequence of Fascin-1 into the pCDH-puro vector (System Biosciences #CD510B-1). The empty pCDH-puro vector was considered as empty vector (EV) control. Myr-Akt1 plasmid (#17245) was obtained from Addgene (USA). To knock down Fascin-1 or HIF-1α, sgRNAs targeting Fascin-1 (sg-Fascin-1#1 and sg-Fascin-1#2) and HIF-1α (sg-HIF-1α#1 and sg-HIF-1α#2) were designed and inserted into the lentiCRISPRv2 vector (Addgene #52961). The lentiCRISPRv2 vector inserted with a non-targeting sequence was used as sg-NC control. The sequence for sgRNAs were listed in Supplementary Table 2.

### Quantitative real-time polymerase chain reaction

Total RNAs from cell lines and tissue samples were extracted using TRIzol reagent (Invitrogen, USA) as protocol indicates. Complementary DNA was synthesized using PrimeScript RT reagent Kit (Takara, Japan). SYBR Premix Ex Taq kit (Takara, Japan) was used for qRT-PCR analysis. Relative gene expression was measured using the 2^−ΔΔCq^ method and normalized to GAPDH. The primers used for qRT-PCR was listed in Supplementary Table 3.

### Transcriptome RNA-sequencing and data analysis

Hep3B cells were exposed to normoxia or hypoxia condition for 24 h, then total RNAs were extracted using TRIzol reagent (Invitrogen, USA) as indicated. RNA was purified by the Ribo-off rRNA Depletion Kit (Human/ Mouse/ Rat)(Vazyme #N406), and cDNA library was prepared using the VAHTS Universal V8 RNA-seq Library Prep Kit for lllumina (Vazyme #NR605) as protocol indicates. Then, cDNA library was sequenced using Illumina HiSeq 2500. The expression levels of genes were calculated using the RPKM values. Dysregulated genes were defined as |log_2_ fold change | ≥ 1 and adjusted *P* < 0.05. The significant affected signaling pathways were evaluated by Kyoto Encyclopedia of Genes and Genomes (KEGG) and gene ontology (GO) analysis using DAVID 6.8 tools. For TCGA and GEO data analysis, the Fascin-1 expression datasets and survival data was downloaded from The Cancer Genome Atlas (TCGA) and GEO (GSE36376, GSE39791, and GSE102079). PRADA tool was used to align the RNA-sequencing data. HTSeq V0.6.1 was used to evaluate the RNA-sequencing reads. Limma package (version: 3.40.2) of R software was used to identify differentially expressed mRNAs. All samples were conducted with three repeats.

### Western blot

Tissue samples or culture cells were lysed by RIPA buffer (Beyotime, China). Cell lysates of the entire cells or nucleus fractions were collected. Protein concentration was measured by BCA kit (Thermo Fisher, USA). In all, 30 μg of protein was separated by 10% or 12% SDS-PAGE and transferred onto PVDF membrane (Bio-Rad, USA). Then, membrane was blocked with 5% non-fat milk and incubated with specific first antibody at 4 °C overnight and second antibody for 1 h at room temperature. The protein band was revealed by ECL plus kit (ThermoFisher, USA) using the ChemiDoc Touch Imaging System (Bio-Rad, USA). The specific first antibodies were listed in Supplementary Table 4.

### Transwell migration and invasion assays

The transwell chamber (Millipore, USA) was used for migration and invasion assays. To evaluate cell migration, cells (2.5 × 10^4^ cells) were seeded in the upper chamber without serum, and the lower chamber was full of RMPI-1640 medium with 10% fetal bovine serum. To evaluate cell invasion, the chamber was pre-coated with Matrigel (BD, USA). Then, cells (2.5 × 10^4^ cells) were seeded in the upper chamber without serum, and the lower chamber was full of RMPI-1640 medium with 10% fetal bovine serum. Cells were allowed to migrate or invade for 24 h, then fixed with 4% paraformaldehyde for 10 min and stained with crystal violet at room temperature. The migration or invasion cells were photographed by a light microscope (Leica, Germany). Five random fields (400×) were selected and the number of migration or invasion cells were counted. All samples were conducted with three repeats.

### Sphere-formation assay and soft agar assay

To evaluate sphere formation, cells (2000) were cultured in DMEM/F12 medium (Hyclone, USA) supplemented with 2% B27, 10 ng/mL EGF and 10 ng/mL FGF in 6-well ultra-low attachment plates (Corning, USA). Next, cells were subcultured at 2000 cells/well every 5 days to develop secondary or tertiary spheres. Soft agar assay was conducted by seeding cells (6000/well) into 0.4% top agar. The bottom agar was 0.6%. Then, cells were cultured for 3 weeks and colonies were stained with 1 mg/mL MTT for 2 h at 37 °C (Sigma, USA). Images were photographed by a microscope (Leica, Germany). All samples were conducted with three repeats.

### Tumor xenograft model

The animal experiments were reviewed and approved by the ethics committee of Affiliated Hospital of Youjiang Medical University for Nationalities. Hep3B cells (2 × 10^6^ cells) introduced with sg-Fascin-1#1, sg-Fascin-1#2 or sg-NC, or HepG2 cells (2 × 10^6^ cells) introduced with Fascin-1 expression lentivirus or EV control, were subcutaneously injected into 6-week-old female nude mice. There were 5 mice for each group of Hep3B cells and 4 mice for each group of HepG2 cells. Tumor volume was measured every 3 days from day 7 after cell injection, and evaluated by the formula (length × width^2^)/2. Four weeks later, mice were anaesthetized by inhalation with 3% isoflourane and sacrificed by broking the neck. Then, tumor xenografts were dissected out and weighed.

### Gelatin zymography

Gelatin zymography was conducted as previously reported [34]. Briefly, cells (1 × 10^5^) were seeded in 24-well plates and cultured with serum-free conditioned medium for 24 h. Then, conditioned medium was concentrated using cold ethanol and dissolved in 50 μL H_2_O. The samples were loaded on 10% SDS-PAGE containing 0.1% gelatin under nonreducing conditions. Then, gels were stained with 0.4% Coomassie blue. Gelatinolytic activities for MMP-2 and MMP-9 were evaluated using dialysis membranes (Amersham, NJ). Each sample was done in triplicates.

### Immunofluorescence

Hep3B cells introduced with sg-Fascin-1#1, sg-Fascin-1#2, or sg-NC, or HepG2 cells introduced with Fascin-1 expression lentivirus or EV control were seeded on coverslips, then fixed with 4% paraformaldehyde for 15 min and permeabilized with 1% Triton X-100 for 15 min. F-actin was stained with Alexa Fluor^®^ 555 Phalloidin (Cell signaling #8953, 1:50) for 15 min at room temperature in Hep3B cells. YAP was stained with YAP Rabbit mAb Alexa Fluor^®^ 488 Conjugate (Cell signaling #14729, 1:50) in Hep3B and HepG2 cells. Nucleus was stained with DAPI (Sigma, USA) for 10 min at room temperature. Cells were washed with PBS once, then images were photographed by a LSM 5 Pascal Laser Scanning Microscope (Zeiss, Germany).

### Cell viability assay

The viability of cells was determined using the Cell Counting Kit-8 (Beyotime, China). Briefly, Hep3B or HuH-6 cells (3000/well) introduced with sg-Fascin-1#1, sg-Fascin-1#2, or sg-NC were seeded in 96-well plates and treated with Sorafenib for 6 days. Then, 10 μL CCK-8 solution was added to each well and incubated for 1 h at 37 °C. The optical density at 450 nm was recorded by a microplate reader. Each sample was done in triplicates.

### Flow cytometry

Cells were dispersed as single-cell suspensions with 0.05% trypsin. A total of 1 × 10^6^ cells were collected and stained with Annexin-V-FITC (Sigma, USA) and propidium iodide (PI) (Sigma, USA) for 15 min at room temperature in a dark room. The signal at 488/530 was determined by a FACS LSR Fortessa (BD Biosciences, USA) and analyzed by FlowJo 10.7 software. Each sample was done in triplicates.

### Statistical analysis

Data was analyzed using GraphPad Prism 8.0 software. Two-tailed Student’s *t* test and one-way ANOVA (LSD post hoc test) were used to compare difference for two or more groups. Overall survival of liver cancer patients was evaluated using Kaplan-Meier plot. Half-maximal inhibitory rate (IC50) of Sorafenib was measured by GraphPad Prism 8.0. Data was shown as mean ± standard deviation (*x* ± s.d.). *P* *<* 0.05 was considered statistically significant.

## Supplementary information

Supplementary Figure 1.

Supplementary Figure 2.

Supplementary Figure legends.

Supplementary Table 1.

Supplementary Table 2.

Supplementary Table 3.

Supplementary Table 4.

Agree to changes authors.

## Data Availability

The datasets used and/or analyzed during the current study are available from the corresponding author on reasonable request.
