# Supplementary figures and images for: Hypoxia-induced Fascin-1 upregulation is regulated by Akt/Rac1 axis and enhances malignant properties of liver cancer cells via mediating actin cytoskeleton rearrangement and Hippo/YAP activation
Source: Cell Death Discov. 2021 Dec 11;7:385. doi: 10.1038/s41420-021-00778-5 (PMC8665929; doi:10.1038/s41420-021-00778-5)

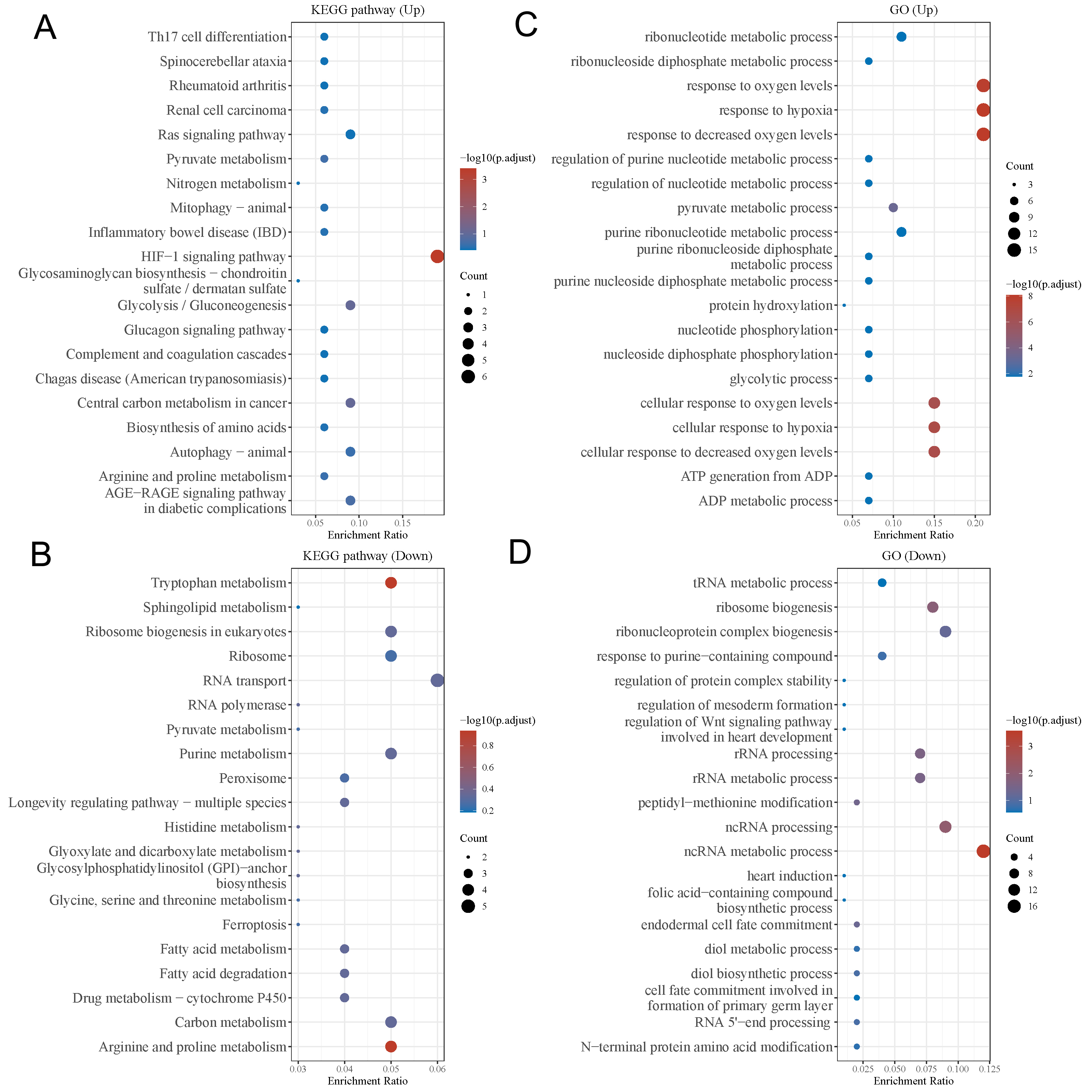

Supplement: Supplementary file 1 — Supplementary Figure 1. [file 41420_2021_778_MOESM1_ESM.jpg]

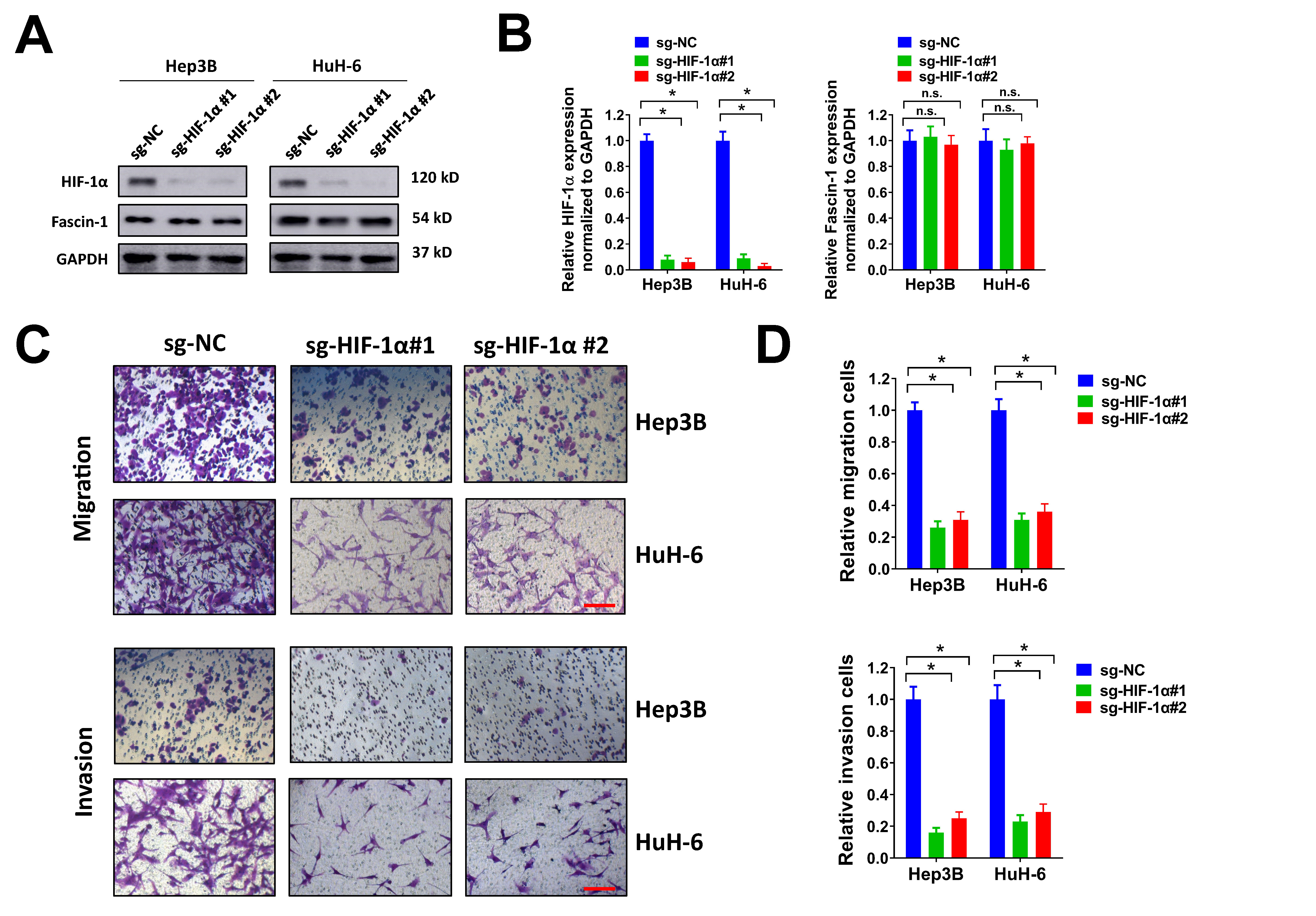

Supplement: Supplementary file 2 — Supplementary Figure 2. [file 41420_2021_778_MOESM2_ESM.jpg]
