# Supplementary material for: Hypoxia-induced Fascin-1 upregulation is regulated by Akt/Rac1 axis and enhances malignant properties of liver cancer cells via mediating actin cytoskeleton rearrangement and Hippo/YAP activation
Source: Cell Death Discov. 2021 Dec 11;7:385. doi: 10.1038/s41420-021-00778-5 (PMC8665929; doi:10.1038/s41420-021-00778-5)
Supplement: Supplementary file 3 — Supplementary Figure legends. [file 41420_2021_778_MOESM3_ESM.docx]

Figure S1. KEEG and GO pathway analysis. The significant affected signaling pathways in Hep3B cells exposed to hypoxia versus normoxia were evaluated by KEGG (A-B) and GO (C-D) analysis using DAVID tools.

Figure S2. HIF1-α knockdown inhibits migration and invasion of liver cancer cells but shows no influence on Fascin-1 levels under hypoxia. A-B, Hep3B or HuH-6 cells infected with sg-HIF-1α#1, sg-HIF-1α#2 or sg-NC lentivirus were exposed to hypoxia condition for 72 h, then collected lysates for western blot (A). Relative Fascin-1 or HIF-1α expression normalized to GAPDH was shown (B). C-D, Hep3B or HuH-6 cells (2.5×10^4^) infected with sg-HIF-1α#1, sg-HIF-1α#2 or sg-NC lentivirus were seeded in transwell chamber for transwell migration or invasion assay under hypoxia condition (C). Relative migration or invasion cells were shown (D). Scale bar = 100 μm. **P＜* 0.05.
