# Supplementary material for: Hypoxia-induced Fascin-1 upregulation is regulated by Akt/Rac1 axis and enhances malignant properties of liver cancer cells via mediating actin cytoskeleton rearrangement and Hippo/YAP activation
Source: Cell Death Discov. 2021 Dec 11;7:385. doi: 10.1038/s41420-021-00778-5 (PMC8665929; doi:10.1038/s41420-021-00778-5)
Supplement: Supplementary file 5 — Supplementary Table 2. [file 41420_2021_778_MOESM5_ESM.docx]

| **Name** | **Sequence** |
| --- | --- |
| sg-Fascin-1#1 | 5’- GGGGTCCACGGTTTCCGCCG-3’; |
| sg-Fascin-1#2 | 5’- CGACCACGCAGGCGTCCTGA-3’ |
| sg-HIF-1α#1 | 5’-TTCTTTACTTCGCCGAGATC-3’ |
| sg-HIF-1α#2 | 5’-CCTCACACGCAAATAGCTGA-3 |
| sg-NC | 5’-ACGGAGGCTAAGCGTCGCAA-3’ |

**Supplementary Table 2. sgRNA sequences.**
