# Supplementary material for: Hypoxia-induced Fascin-1 upregulation is regulated by Akt/Rac1 axis and enhances malignant properties of liver cancer cells via mediating actin cytoskeleton rearrangement and Hippo/YAP activation
Source: Cell Death Discov. 2021 Dec 11;7:385. doi: 10.1038/s41420-021-00778-5 (PMC8665929; doi:10.1038/s41420-021-00778-5)
Supplement: Supplementary file 6 — Supplementary Table 3. [file 41420_2021_778_MOESM6_ESM.docx]

**Supplementary Table 3. Primers of indicated genes used in qRT-PCR.**

| **Gene name** | **Sense primer** | **Anti-senser primer** |
| --- | --- | --- |
| Oct4 | GCAAGCGATCAAGCAGCGACTA | ACCGAGGAGTACAGTGCAGT GA |
| Lin28 | GATTCTCCTGCCTCAGCCTCCT | CCAGCCTGGACAACATGGTG AA |
| Nanog | TGGAGGGTGGAGTATGGTTGGA | AGGCAGGAGAATGGCTGAA C |
| Sox2 | GTACTGGCGAACCATCTCTGTG | TACCAACGGTGTCAACCTGCAT |
| GAPDH | TGGCACCGTCAAGGCTGAGAA | TGGTGAAGACGCCAGTGGAC TC |
| Fascin-1 | TGCCAATCAGGACGAGGAGACC | ATTCTTGCTGGAGGCGGTGGA |
