# Supplementary material for: Hypoxia-induced Fascin-1 upregulation is regulated by Akt/Rac1 axis and enhances malignant properties of liver cancer cells via mediating actin cytoskeleton rearrangement and Hippo/YAP activation
Source: Cell Death Discov. 2021 Dec 11;7:385. doi: 10.1038/s41420-021-00778-5 (PMC8665929; doi:10.1038/s41420-021-00778-5)
Supplement: Supplementary file 7 — Supplementary Table 4. [file 41420_2021_778_MOESM7_ESM.docx]

**Supplementary Table 4. Antibodies used in western blot analysis.**

| **Gene name** | **Company** | **Catalog number** | **Dilutions** |
| --- | --- | --- | --- |
| Fascin-1 | Abcam | ab126772 | 1: 1000 |
| HIF-1α | Cell signaling | 3716 | 1: 1000 |
| GAPDH | Cell signaling | 5174 | 1: 1000 |
| E-Cadherin | Cell signaling | 3195 | 1: 1000 |
| N-cadherin | Cell signaling | 4061 | 1: 1000 |
| Vimentin | Cell signaling | 5741 | 1: 1000 |
| Oct4 | Cell signaling | 2750 | 1: 1000 |
| LIN28A | Cell signaling | 8641 | 1: 1000 |
| Nanog mAb | Cell signaling | 5448 | 1: 1000 |
| Sox2 | Cell signaling | 3579 | 1: 1000 |
| Phospho-Akt | Cell signaling | 4060 | 1: 1000 |
| Akt | Cell signaling | 9272 | 1: 1000 |
| Rac1-GTP | NewEastBio | 26903 | 1: 1000 |
| Rac1 | Abcam | ab155938 | 1: 1000 |
| PTEN | Cell signaling | 9188 | 1: 1000 |
| MMP-9 | Cell signaling | 13667 | 1: 1000 |
| MMP-2 | Cell signaling | 40994 | 1: 1000 |
| YAP | Cell signaling | 14074 | 1: 1000 |
| Phospho-YAP (Ser127) | Cell signaling | 13008 | 1: 1000 |
| LATS1 | Cell signaling | 3477 | 1: 1000 |
| phospho-LATS1 (Thr1079) | Cell signaling | 8654 | 1: 1000 |
| Lamin B1 | Cell signaling | 13435 | 1: 1000 |
