# Supplementary material for: Hypoxia-induced Fascin-1 upregulation is regulated by Akt/Rac1 axis and enhances malignant properties of liver cancer cells via mediating actin cytoskeleton rearrangement and Hippo/YAP activation
Source: Cell Death Discov. 2021 Dec 11;7:385. doi: 10.1038/s41420-021-00778-5 (PMC8665929; doi:10.1038/s41420-021-00778-5)

All co-authors of the article agree to add Youguan Huang and Quan Fang as co-authors for the article entitled “Hypoxia-induced Fascin-1 upregulation is regulated by Akt/Rac1 axis and enhances malignant properties of liver cancer cells via mediating actin cytoskeleton rearrangement and Hippo/YAP activation”, which is submitted to Cell Death Discovery, Manuscript Number: CDDISCOVERY-21-2457R1.

The following is the E-mail reply for all co-authors:


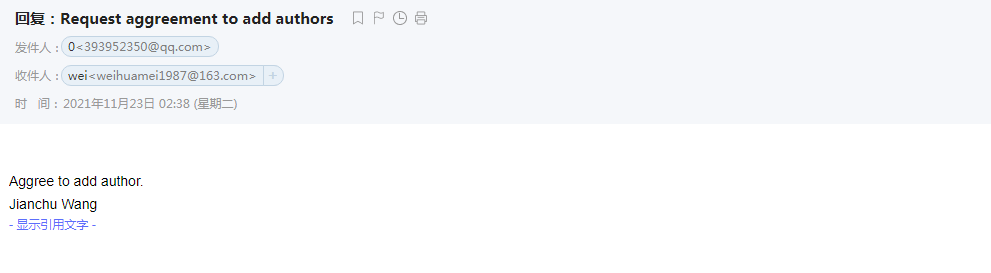


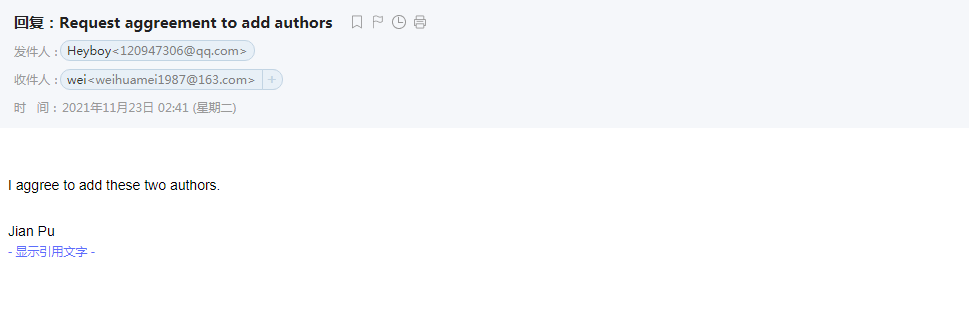


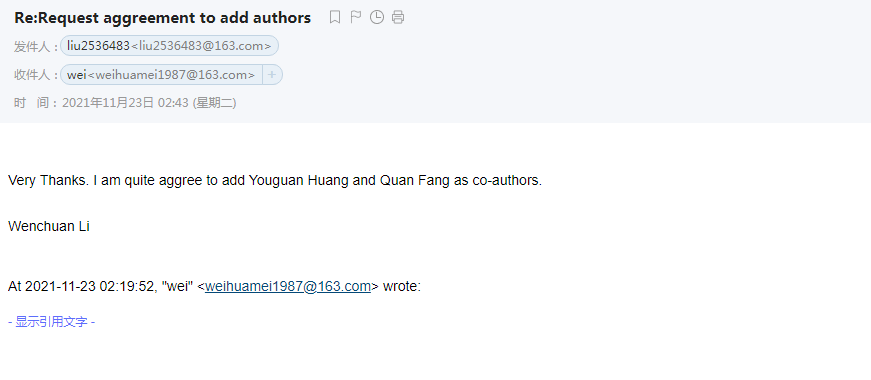


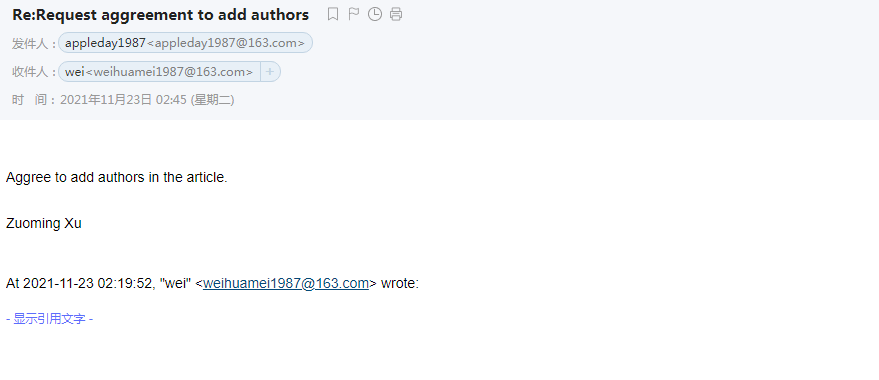


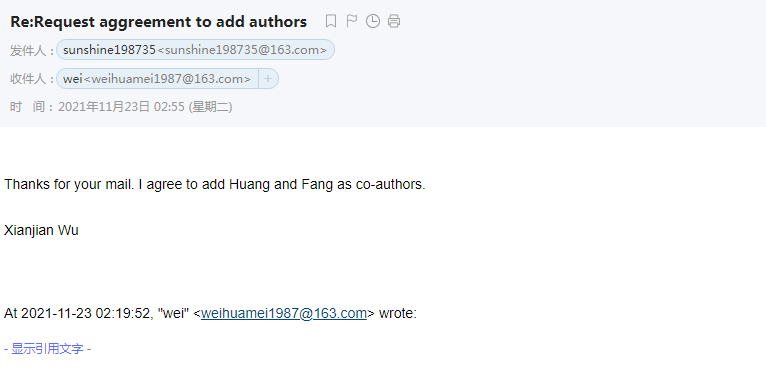


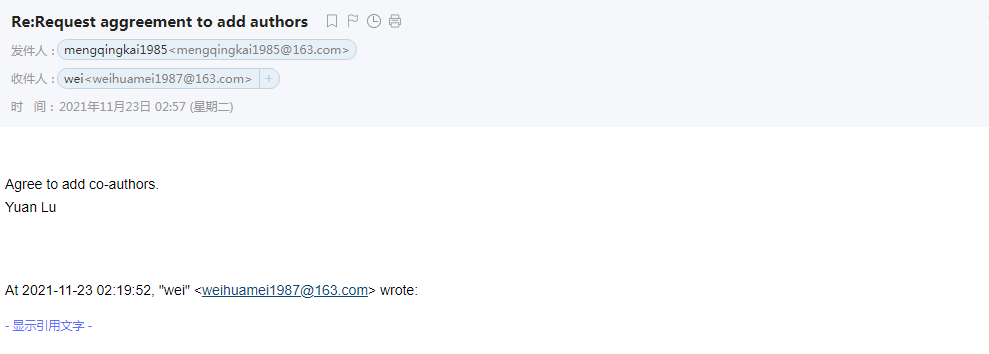


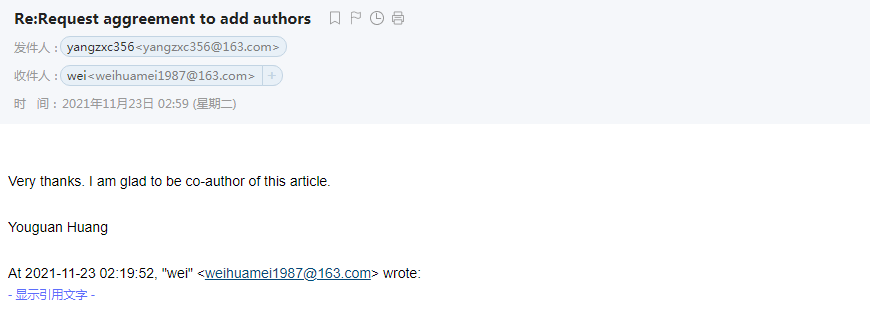


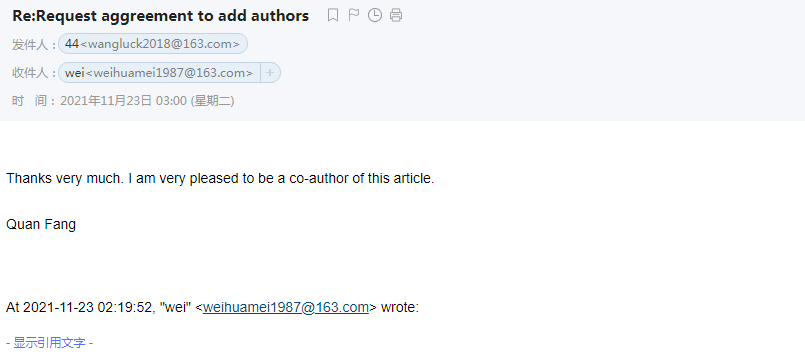

Supplement: Supplementary file 8 — Agree to changes authors. [file 41420_2021_778_MOESM8_ESM.docx]
